# Supplementary material for: Toward the modeling of mucus draining from human lung: role of airways deformation on air-mucus interaction
Source: Front Physiol. 2015 Aug 5;6:214. doi: 10.3389/fphys.2015.00214 (PMC4525672; doi:10.3389/fphys.2015.00214)
Supplement: Supplementary file 1 [file SupplementaryMaterials.PDF]

Appendix A: Computation of pressure drop per unit length - function F

In this appendix, we describe how we compute the pressure drop per unit length in a branch, $C = \partial p/\partial z = F(\Phi_a, S_a, S_b)$, see equation (11). The way we compute the function F is identical to that used in [20]. The expression of F is a consequence of air fluid dynamics and depends on the mucus states.

All calculations take place in a branch of the tree whose radius is $r_b = \sqrt{S_b/\pi}$ and air lumen area radius is $r_a = \sqrt{S_a/\pi}$. The yield stress of mucus is σ_0 and its viscosity when it flows is μ_m. Mucus is solid on the range of radius $[r_a, r_0]$ and liquid on the range $[r_0, r_b]$. If $r_0 \geq r_b$ then mucus is fully solid and if $r_0 \leq r_a$ then mucus is fully liquid. The yield radius r_0 is equal to $\left|\frac{2\sigma_0}{C}\right|$, see [20]. Air viscosity is μ_a.

We call $C = \partial p/\partial z$ the pressure drop per unit length in the branch. The air flow in the branch is known and is equal to Φ_a. Finally, we call s the sign of $C = \partial p/\partial z$ ($s = 1$ if $C \leq 0$ and $s = -1$ if $C < 0$).

Because of developed flow and axi-symmetry hypotheses, fluid dynamics equations reduce to, see [20]:

$$\text{for } r \in [0, r_a],\ -\frac{1}{r}\frac{\partial}{\partial r}\left(r\Sigma_{zr}\right) + \frac{\partial p}{\partial z} = 0 \ \text{ with } \ \Sigma_{zr} = \mu_a \frac{\partial v}{\partial r}$$

By integration on r, the previous equation becomes: $\mu_a \frac{\partial v}{\partial r} = \frac{Cr}{2}$.

The air flow in a branch is equal to: $\Phi_a = 2\pi \int_0^{r_a} v(r) r dr$ and, integrating by parts, it becomes:

$$\Phi_a = 2\pi \left(v(r_a)\frac{r_a^2}{2} - \frac{C}{4\mu_a}\frac{r_a^4}{4} \right)$$

where $v(r_a)$ is the velocity of the air/mucus interface. This velocity depends on the state of mucus.

First state: mucus is liquid between the branch wall located at r_b and the radius r_0 and solid elsewhere (case $r_a < r_0 < r_b$). In that case $v(r_a) = v(r_0)$ since mucus is solid between r_0 and r_a. Then $v(r_a) = v(r_0) = -\frac{C}{4\mu_m}(r_b - r_0)^2$. Replacing $v(r_a)$ with the previous expression in the expression of Φ_a and using the fact that $r_0 = |2\sigma_0/C|$, then C is the solution of a second degree polynomial. The two solutions are:

$$C_1 = \frac{1}{2}\frac{8\pi r_a^2 \mu_a r_b \sigma_0 - 8\phi_a \mu_a \mu_m + 4\sqrt{-8\pi r_a^2 \mu_a^2 r_b \sigma_0 \phi_a \mu_m + 4\phi_a^2 \mu_a^2 \mu_m^2 - 2\pi^2 r_a^6 \mu_a \sigma_0^2 \mu_m}}{\pi r_a^4 \mu_m + 2\pi r_a^2 \mu_a r_b^2}$$

and

$$C_2 = \frac{1}{2}\frac{8\pi r_a^2 \mu_a r_b \sigma_0 - 8\phi_a \mu_a \mu_m - 4\sqrt{-8\pi r_a^2 \mu_a^2 r_b \sigma_0 \phi_a \mu_m + 4\phi_a^2 \mu_a^2 \mu_m^2 - 2\pi^2 r_a^6 \mu_a \sigma_0^2 \mu_m}}{\pi r_a^4 \mu_m + 2\pi r_a^2 \mu_a r_b^2}$$

We have two possible pressure drops per unit length which gives two possible values for $r_0 = |2\sigma_0/C|$, however they are easily discriminated since one only at a time can check $r_b < r_0 < r_a$.

Second state: mucus is liquid everywhere, i.e. $r_0 < r_a$. Then, $v(r_a) = \frac{C}{4\mu_m}(r_a - r_b)(r_a + r_b - 2r_0)$. Mixing with Φ_a expression leads to

$$C = -\frac{8\mu_a}{\pi r_a^2}\frac{\phi_a \mu_m - \pi r_a^3 \sigma_0 + \pi r_a^2 r_b \sigma_0}{r_a^2 \mu_m + 2r_a^2 \mu_a - 2\mu_a r_b^2}$$

Third state: mucus is completely solid ($r_0 > r_a$). In that case $v(r_a) = 0$ and,

$$C = -\frac{16\mu_a \phi_a}{2\pi r_a^4}$$

Th function F is built thanks to these formulas for C. To discriminate between the different possibilities and know the mucus state, we compute for each case the value(s) of C and compute its associated radius $r_0 = |2\sigma_0/C|$. The case is correct only if the position of the radius r_0 relatively to r_a and r_b is compatible with the case hypotheses.

Appendix B: Computation of mucus flow - function G

As for F, the computation of the mucus flow in a branch $\Phi_m = G(C, S_a, S_b)$ depends on the state of mucus, see equation (14). The way we compute the function G is identical to that used in [20]. All calculations take place in a branch of the tree whose radius is $r_b = \sqrt{S_b/\pi}$ and air lumen area radius is $r_a = \sqrt{S_a/\pi}$. The yield stress of mucus is σ_0 and its viscosity when it flows is μ_m. Mucus is solid on the range of radius $[r_a, r_0]$ and liquid on the range $[r_0, r_b]$. If $r_0 \geq r_b$ then mucus is fully solid and if $r_0 \leq r_a$ then mucus is fully liquid. The yield radius r_0 is equal to $\left|\frac{2\sigma_0}{C}\right|$, see [20].

Fluid dynamics of mucus stands in the range $[r_a, r_b]$ and reduces to

$$\begin{array}{ll} \frac{\partial v}{\partial r} = 0 & \text{where mucus is solid (typically in } [r_a, r_0]) \\ \Sigma_{zr} = \frac{Cr}{2} = \sigma_0 + \mu_m \frac{\partial v}{\partial r} & \text{where mucus is liquid (typically in } [r_0, r_b]) \end{array}$$

The flow of mucus is given by

$$\Phi_m = 2\pi \int_{r_a}^{r_b} v(r) r dr$$

Solving fluid dynamics equations for air and mucus then leads to analytical expressions for mucus flow in the branch:

First case: mucus is liquid between the branch wall located at r_b and the radius r_0 and solid elsewhere (case $r_a < r_0 < r_b$). In that case $v(r_a) = v(r_0)$ since mucus is solid between r_0 and r_a. Then $v(r_a) = v(r_0) = -\frac{C}{4\mu_m}(r_b - r_0)^2$, and

$$\Phi_m = 2\pi \frac{v(r_a)}{2}(r_s^2 - r_a^2) - 2\pi \frac{C}{16\mu_m}(r_b^2 - r_s^2)^2$$

Second case: mucus is liquid everywhere, i.e. $r_0 < r_a$. Then, $v(r_a) = \frac{C}{4\mu_m}(r_a - r_b)(r_a + r_b - 2r_0)$, and

$$\Phi_m = -2\pi \frac{C}{16\mu_m}(r_b^2 - r_a^2)^2$$

Third case: mucus is completely solid ($r_0 > r_a$). In that case $v(r_a) = 0$ and $\Phi_m = 0$.

The value of r_0 relatively to r_a and r_b allows to find in which case we are and then to compute the function $\Phi_m = G(C, S_a, S_b)$.

Appendix C: Numerics

The mathematical problem arising from the mechanics are solved using numerical computations. If $v = (v_i)_i$ and $w = (w_i)_i$ are two vectors then we define the product $v \star w$ as the vector $(v_i w_i)_i$. If A is a matrix $n \times n$ and v a vector of length n then the matrix-vector product is written $A.x$. The equations are rewritten in a vectorial form using matrices-vector product . and vector-vector product $\star$:

$$\left\{ \begin{array}{l} S_b = H(P_{ext}, P_{air}) \\ \frac{dS_a}{dt} \star L_b = A.\Phi_a - n_{alv} \star \Phi_{alv} \\ C = F(\Phi_a, S_a, S_b) \\ P_{air} = L.C \\ \left(\frac{dS_b}{dt} - \frac{dS_a}{dt}\right) \star L_b = |\Phi_{m,I}| - |\Phi_{m,O}| \\ \Phi_{m,O} = G(C, S_a, S_b) \\ \Phi_{m,I} = \frac{1}{2}\max(0, U.\Phi_{m,O}) - 2\min(0, D.\Phi_{m,O}) \end{array} \right.$$

Each vector X has its coordinates denoted $X^{(z)}$, for example $S_b = (S_b^{(z)})_z$; the number of vector components is equal to the number of generations considered in the model (23). A, L, U and D are square matrices, see appendix D for details on their expressions. G and F are applied to vectors in this way: $G(C, S_a, S_b) = (G(C^{(z)}, S_a^{(z)}, S_b^{(z)}))_z$ and $F(\Phi_a, S_a, S_b) = (F(\Phi_a^{(z)}, S_a^{(z)}, S_b^{(z)}))_z$.

The equations lead to a non linear vectorial ordinary differential system. It is solved in a full implicit way. Given an initial condition at time 0 and the time-dependence of the tissue pressure $t \to P_{tissue}(t)$, the system is discretized in this way:

$$\begin{cases} S_b(t_n) = H(P_{ext}(t_n), P_{air}(t_n)) \\ \Phi_a(t_n) = A^{-1}\left(\frac{S_a(t_n)-S_a(t_{n-1})}{t_n-t_{n-1}} \star L_b\right) \\ C(t_n) = F(\Phi_a(t_n), S_a(t_n), S_b(t_n)) \\ P_{air}(t_n) = L.C(t_n) \\ S_b(t_n) - S_a(t_n) = S_b(t_{n-1}) - S_a(t_{n-1}) + (t_n - t_{n-1})(|\Phi_{m,I}| - |\Phi_{m,O}|) \star L_b^{-1} \\ \Phi_{m,O}(t_n) = G(C(t_n), S_a(t_n), S_b(t_n)) \\ \Phi_{m,I}(t_n) = \frac{1}{2}\max(0, U.\Phi_{m,O}(t_n)) - 2\min(0, D.\Phi_{m,O}(t_n)) \end{cases}$$

Concretely, we are able to eliminate all -vectorial- variables except $C(t_n)$ and $S_a(t_n)$, thus this system reduces to two non linear vectorial equations:

$$\begin{cases} S_a(t_n) = \mathcal{F}_1(S_a(t_n), C(t_n), S_a(t_{n-1}), S_b(t_{n-1})) \\ C(t_n) = \mathcal{F}_2(S_a(t_n), C(t_n), S_a(t_{n-1})) \end{cases}$$

The system is solved in this way at each time step n:

- in a first step, we build a numerical function $\mathcal{C}_n(S)$ such that $\mathcal{C}_n(S) = \mathcal{F}_2(S, \mathcal{C}_n(S), S_a(t_{n-1}))$. The function $\mathcal{C}_n$ is well defined since $\mathcal{C}_n^{(z)}$ it is a strictly decreasing function of $S^{(z)}$. We use a Newton method to compute $\mathcal{C}_n$, initialized by $C_{init} = L.G(\Phi, S_a(t_n), \mathcal{S}(P_{tissue}(t_n) - P_{air}(t_{n-1})))$ with $\phi = A^{-1}\left(\frac{S-S_a(t_{n-1})}{t_n-t_{n-1}} \star L_b\right)$.
- in a second step, we solve the equation $S = \mathcal{F}_1(S, \mathcal{C}(S), S_a(t_{n-1}), S_b(t_{n-1}))$, again using a Newton method initialized with the air lumen area of the previous time step: $S_{init} = S_a(t_{n-1})$.

We use a parallel Newton method implemented in $C++$ and $OpenMP$. All equations and variables are normalized.

Appendix D: Computation of matrices for the problem vectorial formulation

In this appendix, we give the expression of the matrices A, L, U and D used in the mathematical formulation of the model. In this appendix, the letter i and j refer to generation index and belong to the set $\{0, 1, 2, ..., N-1\}$.

The matrix L relates the pressure drop per unit length to the mean pressure inside the bronchi, see equation (12):

$$L_{i,j} = \begin{cases} 0 & \text{if } j < i \\ l_i/2 & \text{if } j = i \\ l_i & \text{if } j > i \end{cases}$$

The matrix U is used to compute the mucus flow when it goes up in the tree (toward the upper bronchi):

$$U_{i,j} = \begin{cases} 0 & \text{if } j \neq i+1 \\ 1 & \text{if } j = i+1 \end{cases}$$

The matrix D is used to compute the mucus flow when it goes up in the tree (toward the upper bronchi):

$$D_{i,j} = \begin{cases} 0 & \text{if } j \neq i-1 \\ 1 & \text{if } j = i-1 \end{cases}$$

The matrix A relates the volume change of a bronchus with the air flows coming from the two daughter bronchi: $A = Id - 2U$.

Appendix E: Approximation of the Shrek number

In this appendix, we detail how the Shrek number can be approximated with equation (17), starting from its definition in equation (16). First, we need to make the hydrodynamic resistance of an airway in generation i, $R_i = 8\mu_a l_i/(\pi r_i^4)$ appear in the sum:

$$Sh = \frac{1}{N}\sum_{i=0}^{N-1} \frac{4\mu_a \Phi_a^{(i)}}{\pi r_i^3 \sigma_0} = \frac{4}{N\sigma_0}\sum_{i=0}^{N-1} \frac{r_i}{8 l_i} \frac{8\mu_a l_i}{\pi r_i^4} \Phi_a^{(i)}$$

Then, using the fact that the tree is symmetric, we have $\Phi_a^{(i)} = \Phi_a^{(0)}/2^i$, thus:

$$Sh = \frac{1}{2N\sigma_0} \sum_{i=0}^{N-1} \frac{r_i}{l_i} \frac{R_i}{2^i} \Phi_a^{(0)}$$

Because $\Phi_a^{(0)}$ is the flow in the trachea, then it corresponds to the mouth flow Φ_a. The ratio $\frac{r_i}{l_i}$ is approximated by $\frac{1}{6}$ since it is the mean value measured in the lung [29]:

$$Sh = \frac{\Phi_a}{12N\sigma_0} \sum_{i=0}^{N-1} \frac{R_i}{2^i}$$

Finally, the sum $\sum_{i=0}^{N-1} \frac{R_i}{2^i}$ is exactly the equivalent hydrodynamic resistance R_{aw} of our model of the lung with symmetric branching [19, 22]. Finally:

$$Sh = \frac{R_{aw}\Phi_a}{12N\sigma_0}$$
